# Real World Data on the Efficacy of Brigatinib in ALK-Positive Non-Small Cell Lung Cancer: A Single-Center Experience

**DOI:** 10.3390/cancers17183084

**Published:** 2025-09-21

**Authors:** Vesna Ćeriman Krstić, Natalija Samardžić, Mihailo Stjepanović, Spasoje Popević, Tatjana Adžić-Vukičević, Sofija Glumac, Ruža Stević, Dragana Marić, Marta Velinović, Milena Jovanović, Branislav Ilić, Milija Gajić, Nikola Čolić, Katarina Lukić, Brankica Milošević Maračić, Slavko Stamenić, Ivana Sekulović Radovanović, Jelena Milin Lazović

**Affiliations:** 1 Faculty of Medicine, University of Belgrade, 11000 Belgrade, Serbia; natalis.dm@gmail.com (N.S.);; 2 Clinic for Pulmonology, University Clinical Center of Serbia, 11000 Belgrade, Serbia; 3 Institute for Pathology, Faculty of Medicine, University of Belgrade, 11000 Belgrade, Serbia; 4 Center for Radiology, University Clinical Center of Serbia, 11000 Belgrade, Serbia; 5 Institute of Oncology and Radiology Serbia, 11000 Belgrade, Serbia; 6 Institute of Medical Statistics, Faculty of Medicine, University of Belgrade, 11000 Belgrade, Serbia

**Keywords:** NSCLC, ALK-positive, brigatinib, PFS, OS

## Abstract

**Introduction**: Lung cancer remains the leading cause of cancer death among all cancers. The discovery of epidermal growth factor receptor (EGFR) and anaplastic lymphoma kinase (ALK) mutations led to an increased survival rate in patients with locally advanced and metastatic non-small cell lung cancer (NSCLC). Results of the ALTA 1L study showed superior outcomes for patients treated with brigatinib compared to patients treated with crizotinib. **Background**: We conducted research including 23 patients with ALK-positive lung adenocarcinoma who were treated with brigatinib in first or further lines of therapy. The median follow-up was 22 months (4–66 months). **Results**: There were no significant differences in patient population between the first and further lines of treatment regarding sex distribution (*p* = 0.692), smoking status (*p* = 0.554), ECOG performance status (*p* = 1.000), and baseline presence of brain metastases (*p* = 0.862). The response rate was 47.8%, and disease control was 95.6%. The 12-month progression-free survival (PFS) and overall survival (OS) rates were 85.3% and 86.5%, respectively, while the 60-month PFS rate was not reached, and the 60-month OS rate was 27.1%. The mPFS and mOS were 32 months. **Conclusions:** The results of this analysis are promising, as our patients experienced better outcomes compared to those in the ALTA 1L study, which may be attributed to the small sample size. However, the effectiveness of brigatinib has been confirmed in our clinical practice.

## Simple Summary

Lung cancer remains the leading cause of cancer death among all cancers. Results of the ALTA 1L study showed superior outcomes for patients with ALK-positive NSCLC treated with brigatinib compared to patients treated with crizotinib. We conducted research on patients with ALK-positive NSCLC treated with brigatinib in the first and further therapy lines. Response rate was 47.8%, and disease control was 95.6%. The 12-month progression-free survival (PFS) and overall survival (OS) rates were 85.3% and 86.5%, respectively, while the 60-month PFS rate was not reached, and the 60-month OS rate was 27.1%. The mPFS and mOS were 32 months. The results of this analysis are promising, as our patients experienced better outcomes compared to those in the ALTA 1L study.

## 1. Introduction

Despite the significant progress in the treatment of lung cancer, it still represents a major cause of cancer-related death in both genders [1]. The data from “GLOBOCAN” also showed that lung cancer was the most commonly diagnosed cancer in men and the second most diagnosed among women [1].

Non-small cell lung cancer (NSCLC) accounts for approximately 85% of all cases of lung cancer [2]. In the past, median overall survival (mOS) for patients with advanced and metastatic disease treated with a platinum doublet was around 8 months, with an overall response rate of less than 20% [2]. The results were similar regardless of the type of platinum doublet used [2]. Also, patients with ECOG PS 2 had a significantly lower survival rate (3.9 months) compared to patients with ECOG PS 0 (10.8 months) or 1 (7.1 months) [2].

Anaplastic lymphoma kinase (ALK) gene rearrangement can be found in 3–5% of all non-small cell lung cancer (NSCLC) cases [3]. Patients with ALK-positive NSCLC represent a different subpopulation of NSCLC [3]. Typically, it occurs in younger patients who are nonsmokers or have a history of light smoking, and patients usually have adenocarcinoma histology [3].

Further, the results showed that 30–40% of patients with ALK-positive NSCLC had brain metastases at baseline [4,5,6,7,8].

Before the introduction of ALK tyrosine kinase inhibitors (TKIs), the median progression-free survival (PFS) for this group of patients when treated with chemotherapy was 7 months, and their prognosis was poor [3]. The first approved ALK TKI was crizotinib. The PROFILE 1014 study compared the efficacy of crizotinib in the first-line setting with chemotherapy, which was the standard of care before ALK TKI introduction [3,9]. The results showed the superiority of crizotinib regarding response rate, PFS, and quality of life. However, results for OS were similar, probably due to the fact that the crossover rate was 70% [3,9]. This study also included patients with ECOG PS 2 and with treated brain metastases (27% of patients had brain metastases) [3,9]. The common site of progression was the brain, possibly due to the low penetration rate of crizotinib into the brain.

Ceritinib, alectinib, and brigatinib represent the second generation of ALK TKIs. These drugs were developed in order to overcome the inevitable acquired resistance to crizotinib, which usually occurs within 12 months [10].

The results of phase I and II studies demonstrated the efficacy of ceritinib in patients who were not treated with crizotinib and also in patients who developed resistance to crizotinib [11]. Further, the results of phase III studies, ASCEND-4 [6] and ASCEND-5 [12], also showed the superiority of ceritinib compared to chemotherapy in the first and further lines of treatment.

Alectinib, another second-generation ALK TKI, was found to be superior to crizotinib and ceritinib due to higher crossover to the blood–brain barrier [11,13]. It was suggested that this higher crossover is probably due to the fact that crizotinib and ceritinib are targets of *p*-glycoprotein, a transmembrane protein that pumps xenobiotics out of the central nervous system, in contrast to alectinib [14]. Further, the studies showed that alectinib has a significantly higher concentration level and penetration rate (86%) in the cerebrospinal fluid compared to crizotinib [15,16,17].

The ALEX trial compared alectinib to crizotinib in the first-line setting, and results showed the superiority of alectinib, with a significantly higher response rate, PFS, OS, and with a lower incidence of CNS progression compared to crizotinib [7]. This result is particularly important since 40% of patients had baseline brain metastases [7].

Brigatinib is an oral second-generation ALK TKI that has been proven to be active against the majority of known resistant mutations [18]. The results of the ALTA 1L study showed the superiority of brigatinib compared to crizotinib. After those results, brigatinib was approved for the treatment of ALK-positive patients [19].

However, at some time point, the acquired resistance also occurs with the second generation of ALK TKI. Lorlatinib, the third generation of ALK TKIs, is active against the majority of known on-target resistance mutations after treatment with second-generation ALK TKIs [20]. Anyhow, the best way to make a decision about further treatment is to discover which resistance mechanism occurred by repeating the biopsy and performing next-generation sequencing [20].

The results of the CROWN study, which compared lorlatinib and crizotinib in the first-line setting, demonstrated the superior efficacy of lorlatinib compared to crizotinib [21,22]. After five years, PFS for patients treated with lorlatinib was not reached, compared to 9.1 months for patients treated with crizotinib [21,22].

The discovery of ALK gene rearrangement and subsequent ALK TKIs significantly improved PFS, OS, and quality of life in this group of patients [18].

The aim of this observational study was to investigate the efficacy of brigatinib in the real-world setting in patients with locally advanced and metastatic ALK-positive NSCLC.

## 2. Materials and Methods

### 2.1. Patients and Data Collection

We enrolled 23 patients treated with brigatinib who had locally advanced or metastatic NSCLC with ALK rearrangement. All included patients were treated in the Clinic for Pulmonology, University Clinical Center of Serbia. All patients are treated until progression of disease or death, whichever happens first. Every patient satisfied the requirements for inclusion: histopathologically confirmed NSCLC with ALK rearrangement positive finding (ALK positivity was determined by Ventana (D5F3) immunohistochemistry assay), locally advanced or metastatic stage of disease, measurable disease at baseline, and patients who were not treated previously with ALK TKIs (some of them were treated with chemotherapy previously). Exclusion criteria—histopathologically verified other subtypes of lung carcinoma, patients who had ALK-negative NSCLC, and early stage of disease. All patients underwent computed tomography of the chest and either computed tomography or magnetic resonance imaging of the head to establish the stage of disease at baseline. We collected data about gender, age, smoking status [i.e., smokers, non-smokers, and ex-smokers (patients who quit smoking a year prior to treatment)], stage of the disease, response to treatment (ORR), PFS, and OS. Response to treatment was defined as complete response (CR), partial response (PR), stable disease (SD), or progression of disease (PD). PFS was evaluated from the start of the treatment with brigatinib until disease progression or death, and OS was evaluated from the start of the treatment with brigatinib until death from any cause. This non-interventional, observational study was conducted in accordance with the Declaration of Helsinki.

### 2.2. Statistical Analysis

Depending on the data type, the results are expressed as means ± standard deviation or count (%). Given the small sample size (*n* = 23), the study was not formally powered to detect statistical differences between groups. The groups were compared by using a parametric t test and the nonparametric Pearson Chi-square test. The Kaplan–Meier with Log-rank test was used to evaluate survival and group differences. Censoring in the survival analysis was handled by treating patients without an event at the end of follow-up as censored, in accordance with standard Kaplan–Meier methodology. Hypothesis testing was reported, but these results should be interpreted cautiously, as *p*-values are of limited value in this context. The median (95% CI) or percentage of participants who did not experience an event of interest within a certain time period is used to display survival. Every *p*-value below 0.05 has been deemed significant. We analyzed all data using SPSS 29.0 (IBM Corp. Released 2023. IBM SPSS Statistics for Windows, Version 20.0. Armonk, NY, USA: IBM Corp.).

## 3. Results

A total of 23 patients with lung adenocarcinoma were included in the analysis, of whom 18 were treated with brigatinib in the first-line setting, and 5 patients were treated with brigatinib in further therapy lines (previously treated with chemotherapy). Baseline demographic characteristics are presented in Table 1.

The median follow-up time for the whole group was 22 months (ranging from 4 to 66 months). The median age for the whole group was 58.4 ± 16 years.

Table 2 presents baseline demographic characteristics by subgroups according to the treatment line in which brigatinib was applied. There were no statistically significant differences between groups. The median follow-up time for patients treated with brigatinib in the first line was 16 months (ranging from 4 to 66 months), and in further therapy lines, 33 months (ranging from 32 to 64 months). The median age for patients treated in the first-line setting was 59.7 ± 16 years, and for patients treated in further therapy lines, it was 53.6 ± 12 years.

The response rate was 47.8% for the whole group of patients, and the disease control rate (DCR) was 95.6%. The data for the entire group of patients, as well as according to the therapy line, are presented in Table 3.

**Figure 1:**
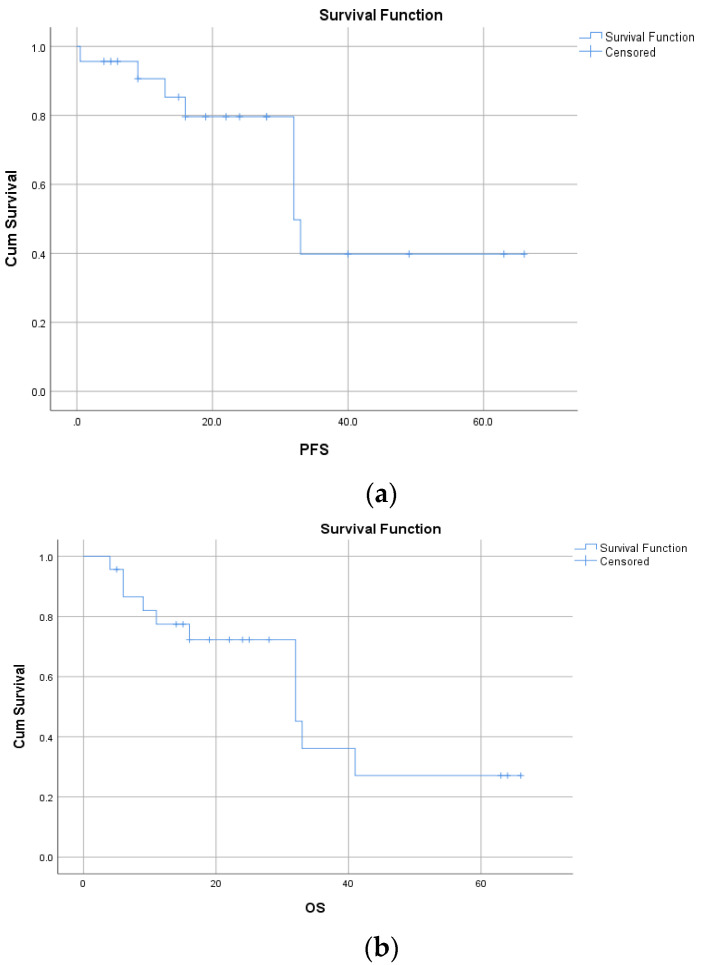
(**a**) The Kaplan–Meier curve for mPFS for whole group of patients, displayed in months (32.0 months), and (**b**) the Kaplan–Meier curve for mOS for all patients treated with brigatinib, displayed in months (32.0 months).

The mPFS for the whole group was 32.0 months (95% CI: 19.6–44.4 months), and the mOS was 32 months (95% CI: 19.3–44.7 months) (Figure 1a,b).

Further, 12-month PFS and OS rates were 85.3% and 77.4%, respectively, and 5-year PFS and OS rates were not reached and 27.1%, respectively. The rates of PFS and OS are presented in Table 4.

**Figure 2:**
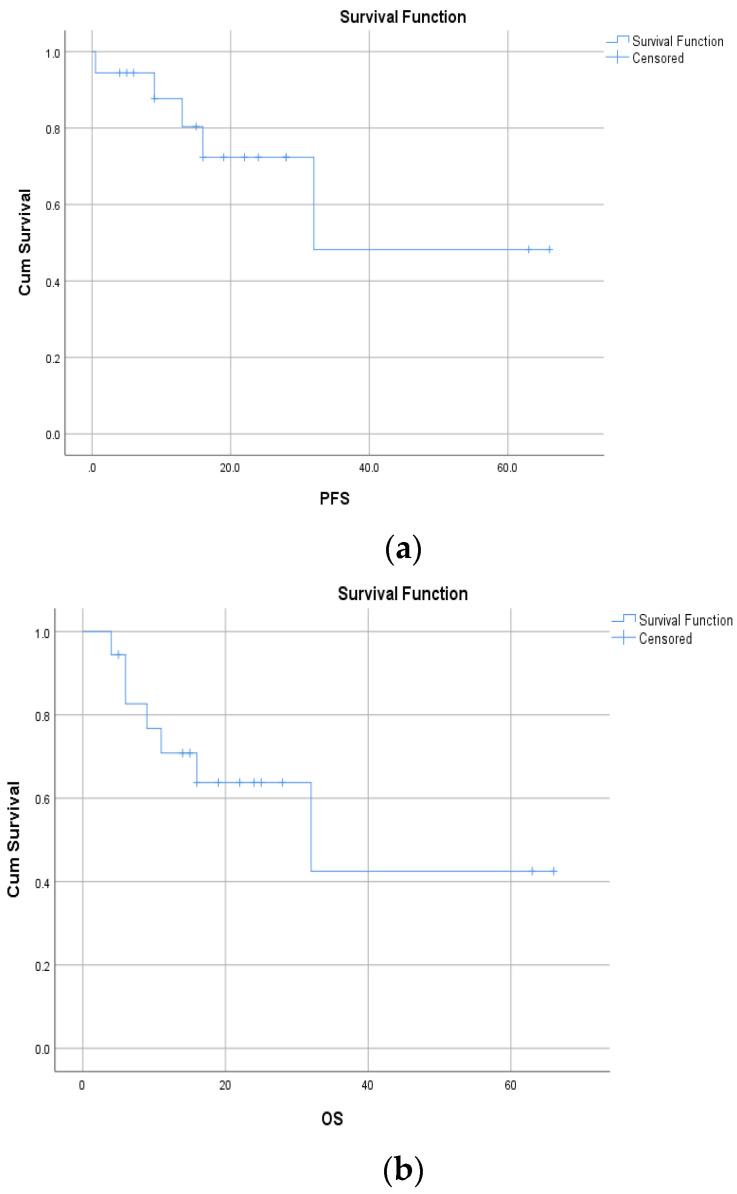
(**a**) The Kaplan–Meier curve for mPFS for patients treated with brigatinib in first-line setting, displayed in months (44.8 months), and (**b**) the Kaplan–Meier curve for mOS for patients treated with brigatinib in first-line setting, displayed in months (32.0 months).

In the first-line setting, mPFS was 44.8 months (95% CI: 27.5–62.1) and mOS was 32 months (95% CI: 3.8–60.2) (Figure 2a,b).

The 12-month PFS and OS rates for patients treated with brigatinib in the first-line setting were 72.4% and 63.8%, respectively, and the 5-year PFS and OS rates were 48.2% and 42.5%, respectively. In Table 5, PFS and OS rates are presented for patients treated with brigatinib in the first-line setting.

**Figure 3:**
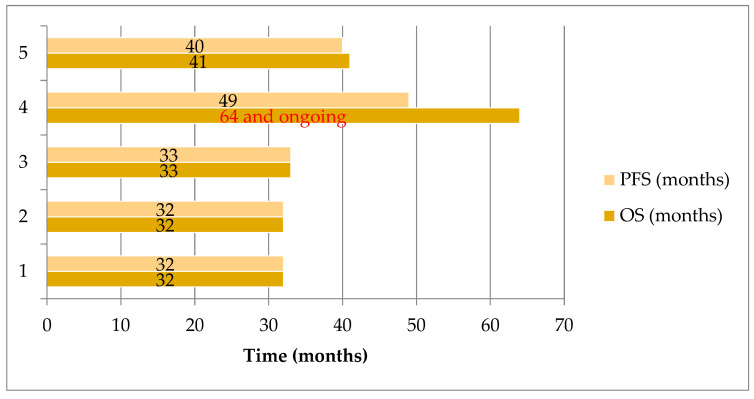
PFS and OS for each of 5 patients treated with brigatinib in further therapy lines, displayed in months (39.0 and 33.0 months, respectively).

The mPFS in further lines was 39 months (95% CI: 31.8–46.2 months) and mOS was 33 months (95%CI: 30.9–35.1 months). Since there were only five patients treated with brigatinib in further therapy lines, we presented PFS and OS results for each patient separately (Figure 3).

## 4. Discussion

After the results of the ALTA 1L study [19], brigatinib found its place in the first-line treatment for patients with locally advanced and metastatic NSCLC. This study investigated the efficacy of brigatinib in ALK-positive locally advanced and metastatic NSCLC. The median follow-up was 40 months. Five percent of patients in the brigatinib arm had ECOG PS 2, and the majority of included patients had a metastatic stage of disease (94%) [19]. The majority of patients had non-squamous histology (97%), and 29% of patients treated with brigatinib had baseline CNS metastases [19]. Further, 26% of patients received chemotherapy in a first-line setting [19]. The 12-month PFS rate was 67%, and the confirmed ORR was 71% [23]. The 3-year PFS rate was 43%, and mPFS was 24 months [19]. The 3-year OS rate was 71%, and mOS was not reached [19]. In a group of patients who crossed over to brigatinib after progression on crizotinib, mPFS was 16.8 months [19]. Further, the efficacy of brigatinib was similar in Asian and non-Asian patients [24]. The design of our study was very similar to ALTA 1L (4.3% of patients had ECOG PS 2, 21.7% had brain metastases, and 21.7% of patients were previously treated with chemotherapy), but our patients experienced better outcomes compared to the registration study. However, our group of patients was small, but the efficacy of brigatinib was confirmed in a real-world setting.

The phase 2 J-ALTA trial [25] investigated the efficacy of brigatinib in previously treated and treatment-naïve patients. It included 47 patients who were previously treated with ALK TKIs and 32 treatment-naïve patients. ORR was 34%, and mPFS 7.3 months [25]. In a group of patients who were treatment-naïve, the 2-year PFS rate was 73%, and the ORR was 97% [25].

In the integrated analysis of the ALTA 1L and J-ALTA, there were 137 patients from ALTA 1L and 32 from J-ALTA who were included in the analysis, and they were treatment-naïve [26]. The median age was 58 years, and 5% of patients had ECOG PS 2 [26]. Almost all of the patients were nonsmokers or ex-smokers, and brain metastases were present in 28% of included patients at baseline [26]. The majority of patients, 95% of them, had a metastatic stage of disease at baseline, and prior to chemotherapy, 26% of patients had a metastatic stage of disease [26]. The mPFS was 29.3 months, and the 2- and 3-year PFS rates were 55% and 46%, respectively [26]. ORR was 79%, and DCR was 86% [26]. The 2- and 3-year OS rates were 79% and 74%, respectively [26].

A study conducted by Jeon et al. [27] evaluated the efficacy of alectinib or brigatinib in a first-line setting in 208 ALK-positive patients; 32 patients received brigatinib. The mean age was 57.17 years. The majority of patients were nonsmokers or ex-smokers, and one quarter of patients had brain metastases at baseline [27]. All patients treated with brigatinib had ECOG PS 0-1. The ORR was 93.8% in the brigatinib group [27]. The 12-month PFS and OS rates were 84.1% and 95.2%, respectively [27]. mPFS and mOS were not reached [27].

The Table 6 shows comparative view of some studies that investigated the efficacy of brigatinib.

UVEA-Brig was a retrospective study that evaluated the efficacy of brigatinib in previously treated ALK-positive NSCLC (1 to 6 previous therapy lines) in four different countries with a median follow-up of 16.5 months [28]. Two-thirds of patients had brain metastases, and 15% of patients had ECOG PS 2 and 3 [28]. The RR was 39.8% [28]. The mPFS and mOS were 11.3 months and 23.3 months, respectively [28].

A large multicentric study evaluated the efficacy of brigatinib in 604 previously treated patients with NSCLC [29]. The median time to treatment discontinuation (TTD) for the whole group of patients was 8.7 months, and the data showed that TTD was dependent on the number of the previous treatment lines: 11.8 months, 10.8 months, and 7.7 months in first, second, and further treatment lines, respectively [29].

In a study by Hochmair et al. [30] that included 35 previously treated patients, RR was 84.9%, and mPFS was 9.9 months.

Singh et al. [31] conducted very interesting research in which they analyzed the outcomes in patients with ALK-positive NSCLC who had ECOG PS 2-4. One-quarter of patients had brain metastases at baseline [31]. They found that patients with ECOG PS 2-3 had worse PFS and OS compared to patients with ECOG PS 0-1, and also they found that patients with ECOG PS 2 had better outcomes compared to patients with ECOG PS 3-4 [31]. Further, the results also showed that patients who were treated upfront with ALK TKIs had better outcomes compared to those who were initially treated with chemotherapy [31].

Pike et al. [32] investigated whether upfront stereotactic radiosurgery (SRS) had an impact on outcomes in patients with EGFR and ALK-positive NSCLC. It was shown that time to CNS progression and local CNS control were improved, but without an impact on OS [32]. Also, it was found that patients with brain metastases larger than 1 cm may benefit the most from upfront SRS [32].

A large meta-analysis analyzed intracranial ORR and DCR in over 1000 patients with ALK-positive NSCLC and brain metastases [33]. The results showed that ALK TKIs were effective in all settings, including first and further therapy lines. Intracranial ORR and DCR were similar regardless of treatment line [33]. Also, the efficacy of ALK TKIs was independent of previous radiotherapy [33].

According to current ESMO [34] and NCCN [35] guidelines, patients with oncogene addicted NSCLC should be treated with TKIs, regardless of ECOG PS, since ORR is high and toxicity is manageable.

The limitations of our study include the small number of patients. With only 23 patients, the use of *p*-values does not provide meaningful inference, and the results should be interpreted as descriptive observations rather than hypothesis-driven outcomes. Focusing on confidence intervals and median survival estimates provides a more appropriate representation of the data. Furthermore, the small sample size and single-center design limit the generalizability of the findings. The study population in our study was very similar to ALTA 1L. However, the mPFS in our study was much better compared to ALTA 1L and other presented studies, as we already mentioned; the reason could be the small sample size. The results for OS and PFS were similar in our study, possibly due to the lack of sequencing in our country and a lack of lorlatinib. Lorlatinib is not covered by insurance, and it is also not possible to make a rotation of ALK TKIs, since NGS is not available; thus, we do not know which resistance mechanisms occurred. Also, in some patients, it could be possible that a flare phenomenon had occurred (which is well known in some patients treated with TKIs—after the discontinuation of TKIs, fast progression occurs) [36], since the majority of patients who progressed died soon after the discontinuation of brigatinib.

## 5. Conclusions

The discovery of ALK TKIs created a revolution in the treatment of patients with ALK-positive NSCLC. The results of this real-world analysis are promising, as our patients experienced better outcomes compared to those in the ALTA 1L study, a registration study for brigatinib, which may be attributed to the small sample size. However, the effectiveness of brigatinib was confirmed in our clinical practice.

## Figures and Tables

**Table 1:** Baseline demographic characteristics of the treated patients.

Patient’s Characteristics	Whole Group *n* (%)
Gender (F vs. M)	11 (47.8) vs. 12 (52.2)
Smoking status (current vs. ex vs. nonsmoker)	2 (8.7) vs. 8 (34.8) vs. 13 (56.5)
ECOG PS (0 vs. 1 vs. 2)	2 (8.7) vs. 20 (87) vs. 1 (4.3)
IIIb/c vs. IV	3 (13) vs. 20 (87)
CNS metastases	5 (21.7)
Line of therapy (1st vs. further)	18 (78.3) vs. 5 (21.7)

Abbreviations: F = female; M = male; CNS = central nervous system.

**Table 2:** Baseline demographic characteristics of treated patients according to the treatment lines.

Therapy Line	First Line (*n* = 18)	Further Lines (*n* = 5)	*p*
Gender (F vs. M)	9 (50)/9 (50)	2 (40) vs. 3 (60)	0.692
Smoking status (current vs. ex vs. non)	1 (5.5) vs. 7 (39)vs. 10 (55.5)	1 (20) vs. 1 (20) vs. 3 (60)	0.554
ECOG PS (o vs. 1 vs. 2)	2 (11.1) vs. 15 (83.3) vs. 1 (5.5)	0 (0) vs. 5 (100) vs. 0(0)	1.000
IIIb/c vs. IV	3 (16.7) vs. 15 (83.3)	0 (0) vs. 5 (100)	
CNS metastatis	3 (16.7)	1 (20)	0.862

Abbreviations: F = female; M = male; CNS = central nervous system.

**Table 3:** Response to brigatinib for the whole group of patients and according to the treatment line.

Response	Whole Group (*n* = 23) No. (%)	1st Line (*n* = 18) No. (%)	Further Lines (*n* = 5) No. (%)
CR	2 (8.7)	1 (5.6)	1 (20)
PR	9 (39.1)	8 (44.4)	1 (20)
CR + PR	11 (47.8)	9 (50)	2 (40)
SD	11 (47.8)	8 (44.4)	3 (60)
DCR (CR + PR + SD)	22 (95.6)	17 (94.4)	5 (100)
PD	1 (4.3)	1 (5.6)	0 (0)

Abbreviations: CR = complete response; PR = partial response; SD = stable disease; DCR = disease control rate; PD = progressive disease.

**Table 4:** The PFS and the OS rates for whole group of patients.

Time (Months)	PFS Rate	OS Rate
6-month	90.6%	86.5%
12-month	85.3%	77.4%
24-month	79.6%	72.3%
36-month	39.8%	36.1%
60-month	Not reached	27.1%

Abbreviations: PFS = progression free survival; OS = overall survival.

**Table 5:** The PFS and the OS rates for patients treated with brigatinib in first-line setting.

Time (Months)	PFS Rate	OS Rate
6 months	87.7%	82.6%
12 months	72.4%	63.8%
24 months	72.4%	63.8%
36 months	48.2%	42.5%
60 months	48.2%	42.5%

Abbreviation: PFS = progression-free survival; OS = overall survival.

**Table 6:** Comparative view of some studies that investigated the efficacy of brigatinib.

Study	Treatment Line	PFS (Months)	OS (Months)	12-Month PFS Rate (%)	12-Month OS Rate (%)
ALTA 1L [19,23]	1st and previously treated	24	Not reached	67%	/
J-ALTA [25]	1st and previously treated	7.3	/	/	/
Jeon et al. [27]	1st	Not reached	Not reached	84.1	95.2
UVEA-Brig [28]	Previously treated	11.3	23.3	/	/
Huamao et al. [29]	Previously treated	8.7	/	/	/
Hochmair et al. [30]	Previously treated	9.9	/	/	/
Ceriman Krstic and Samardzic et al.	1st and previously treated	32	32	85.3	77.4

## Data Availability

Data are contained within the article.
